# Incidental intraoperative discovery of a Wolffian tumor in a postmenopausal woman: a case report and literature review

**DOI:** 10.3389/fonc.2025.1674008

**Published:** 2025-10-08

**Authors:** Dong-mei Li, Ming-rong Xi, Xi Zeng

**Affiliations:** ^1^ Department of Obstetrics and Gynecology, West China Second University Hospital, Sichuan University, Chengdu, China; ^2^ Key Laboratory of Birth Defects and Related Diseases of Women and Children (Sichuan University), Ministry of Education, Chengdu, China

**Keywords:** FATWO, diagnosis, differential, laparoscopy, single-site, treatment, case reports

## Abstract

**Background:**

Female adnexal tumor of Wolffian origin (FATWO) is an exceedingly rare gynecologic neoplasm characterized by nonspecific clinical manifestations and diagnostic challenges. This article presents a case of an incidentally discovered FATWO in a postmenopausal woman, with a literature review.

**Case presentation:**

A 54-year-old postmenopausal woman was found to have a 5.2-cm solid mass in the left adnexa during routine examination. Ultrasonography revealed a well-circumscribed lesion with detectable blood flow, while computed tomography (CT) scan suggested an ovarian origin. Tumor markers were within normal limits, and the patient remained asymptomatic. Single-port laparoscopic exploration identified a 5-cm solid ovarian nodule with an intact capsule. Intraoperative frozen section analysis suggested FATWO, prompting subsequent total hysterectomy with bilateral salpingo-oophorectomy. No adjuvant therapy was administered postoperatively, and the patient showed no evidence of disease progression during 1-year follow-up.

**Conclusion:**

FATWO exhibits a potentially malignant biological behavior. For postmenopausal patients, total hysterectomy with bilateral salpingo-oophorectomy is recommended. Postoperative management should be individualized. Current evidence regarding treatment strategies for reproductive-age patients remains limited, warranting further investigation to optimize clinical decision-making.

## Introduction

The Wolffian tumor, also known as the female adnexal tumor of Wolffian origin (FATWO), is a rare and distinctive epithelial neoplasm originating from the Wolffian duct. This pathology was first described by Kariminejad and Scully in 1973 (1). The majority of FATWOs are located in the broad ligament or the mid-portion of the fallopian tube, with only 20% found in the ovarian hilum near the rete ovarii. Patients’ ages vary from 15 to 87 years. Lower abdominal pain/bloating is the most common sign, and a pelvic mass or abnormal genital bleeding is discovered incidentally (2, 3). The rarity of FATWO and its nonspecific clinical presentation and heterogeneous histopathological features make its diagnosis challenging. While the majority of FATWOs exhibit a low malignant potential, aggressive behavior—including recurrence and metastasis—has been reported in rare cases (1, 4). Currently, there is no standardized treatment protocol for FATWO. Here, we present a case of an incidentally discovered Wolffian tumor in a postmenopausal woman during surgery. This report aimed to draw attention to this disease and to explore the clinical management of Wolffian tumors and discuss therapeutic options, contributing to the limited evidence on the management of this rare entity.

## Case presentation

**Figure 1:**
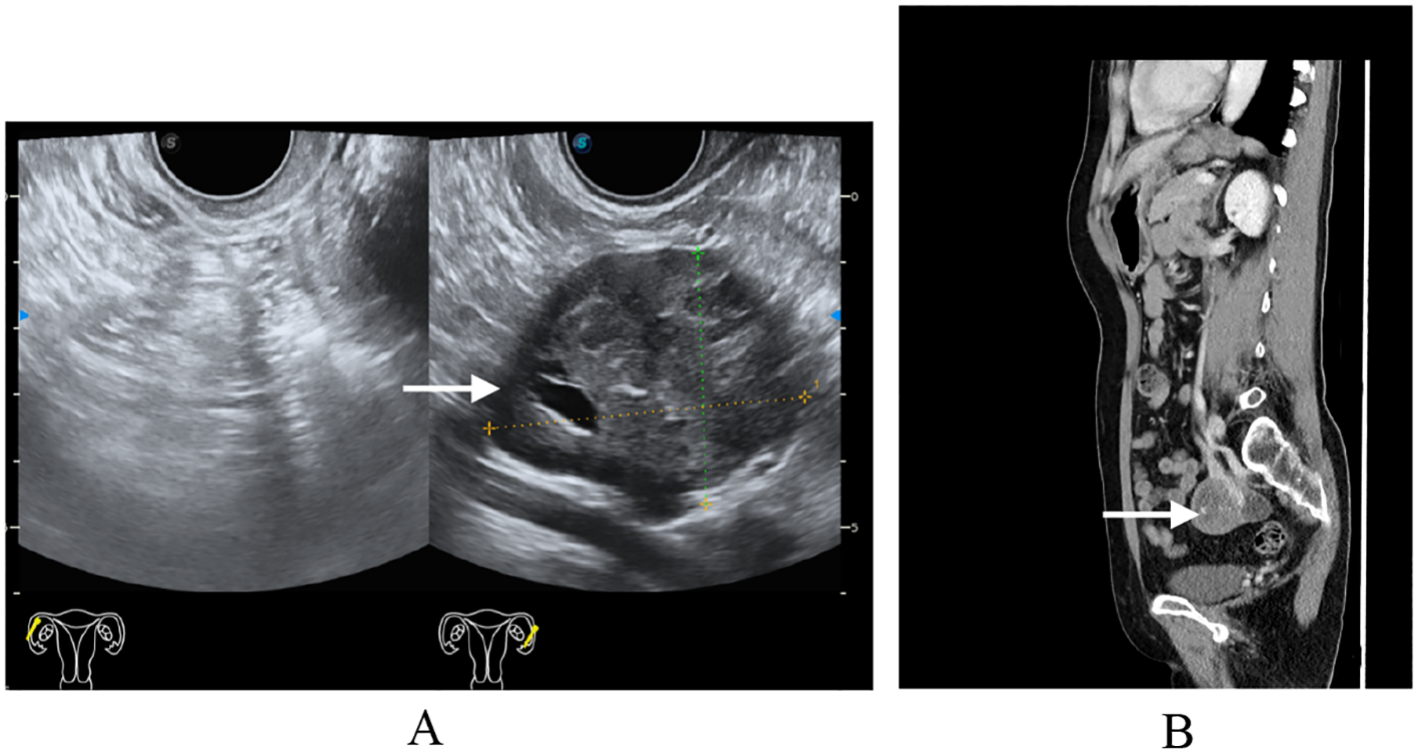
Preoperative imaging findings. **(A)** Transvaginal ultrasound: single-chamber anechoic cystic lesion, 5 cm in diameter, arising from the left adnexa; wall thin and smooth, no internal septa or papillary projections, color Doppler shows absent vascularity. **(B)** Computed tomography same-level non-contrast CT reveals a well-circumscribed, oval low-density cystic mass in the left adnexal region, homogeneous attenuation, no enhancement, adjacent structures mildly displaced without mass effect.

A 54-year-old postmenopausal woman, gravida 3 para 1 (G3P1) with a history of one full-term vaginal delivery and two remote-induced abortions (over 20 years ago), was admitted to our hospital following a routine physical examination in June 2024. She had no significant medical or surgical comorbidities. The patient was asymptomatic, with no abdominal discomfort, abnormal vaginal bleeding, or urinary or gastrointestinal symptoms. Her previous menstrual history was unremarkable. A comprehensive gynecological examination was conducted. Speculum examination revealed no vaginal wall lesions and a normal-appearing cervix (cervical cytology was obtained, with the results within normal limits). Bimanual examination palpated a well-defined, mobile, non-tender, approximately 5-cm mass in the left adnexa. Transvaginal ultrasonography confirmed a corresponding 5.2-cm solid left adnexal mass, demonstrating well-demarcated but irregular margins, heterogeneous echogenicity, and punctate vascular signals on color Doppler. To further characterize the lesion, contrast-enhanced computed tomography (CT) of the abdomen and pelvis was performed, which identified a solid left adnexal mass with heterogeneous enhancement and no evidence of lymphadenopathy (**Figure 1**). The imaging findings were most consistent with an ovarian neoplasm. The tumor markers were within normal limits, including CA-125 (16.7 U/ml; normal range, <35 U/ml), alpha-fetoprotein (AFP) (2.1 ng/ml; normal range, <8.1 ng/ml), and carcinoembryonic antigen (CEA) (0.9 ng/ml; normal range, <2.5 ng/ml for non-smokers). Based on the comprehensive evaluation, including the physical examination, imaging features (well-demarcated borders and absence of ascites or lymphadenopathy), and notably negative tumor markers, the mass was preoperatively assessed as highly likely to be benign. The leading differential diagnoses included an ovarian fibroma/thecoma or a pedunculated uterine leiomyoma. The patient was subsequently scheduled for surgical intervention for both definitive diagnosis and treatment. After the preoperative evaluation and signing of informed consent, the patient received a single-port transumbilical laparoscopic exploration. The surgery confirmed an atrophic uterus, and the left ovary was enlarged, containing a 5-cm × 4-cm × 4-cm solid nodular tumor. The left fallopian tube and the right adnexa were unremarkable. No ascites or peritoneal lesions were detected. Frozen section pathology revealed tumor cells arranged in tubular with solid patterns, suggesting FATWO, although sex cord–stromal tumors remained in the differential diagnosis. After negotiating with the patient’s relatives during the operation, a subsequent total hysterectomy with bilateral salpingo-oophorectomy was performed. The patient recovered uneventfully and was discharged home 4 days later.

**Figure 2:**
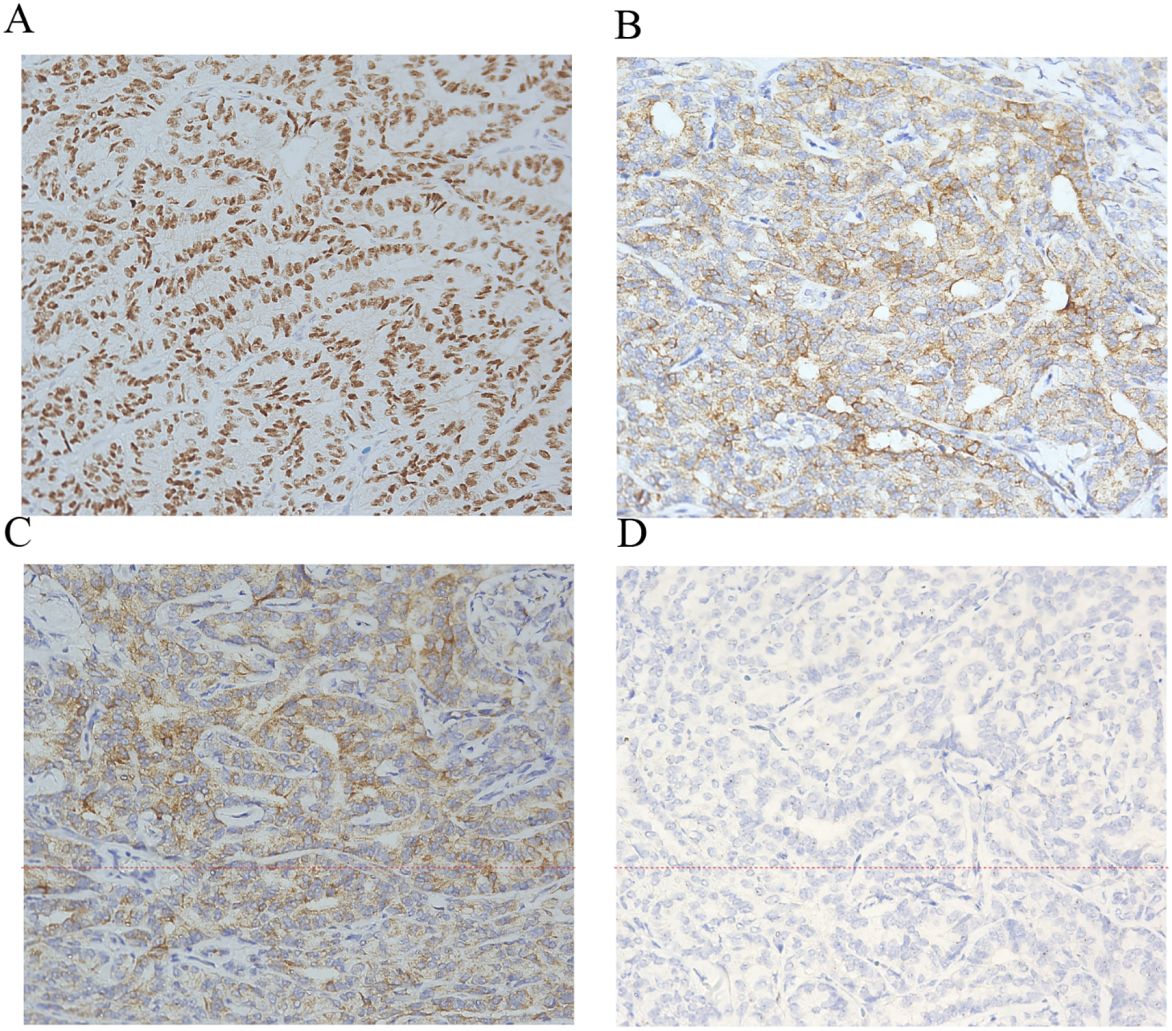
Immunohistochemistry of Wolffian tumor. Immunohistochemical stains (×200) for FOXL2+ **(A)** α-inhibin+ **(B)** calretinin+ **(C)** and epithelial membrane antigen− **(D)**.

Definitive pathology confirmed Wolffian tumor (ICD code: 9110/1), which measured 5.2 cm in maximal diameter. The tumor was confined to the left ovarian parenchyma without capsular invasion, with negative margins, and without involvement of adjacent structures. The resected tumor specimen was thoroughly examined. Macroscopically, the tumor appeared well circumscribed, with a solid and partially cystic cut surface. Microscopically, the tumor exhibited a mixed growth pattern, including solid, tubular, and sieve-like structures. The tumor cells were uniform and bland, with scant cytoplasm and round to oval nuclei. Focal areas showed hyalinized vascular stroma and intraluminal eosinophilic secretions. Immunohistochemistry showed the following: FOXL2 (+++, strongly positive), calretinin (++), SF-1 (++), α-inhibin (++), vimentin (++), CD10 (focal luminal positivity), and AR (+). On the other hand, ER, PR, EMA, GATA3, and TTF-1 were negative. The Ki-67 proliferation index was approximately 1% (**Figure 2**), indicating low cellular activity. These features supported diagnosis as a rare adnexal tumor of borderline malignant potential. Multidisciplinary consensus recommended no adjuvant therapy. A rigorous surveillance protocol was suggested, including quarterly gynecological exams, tumor marker assessments, and imaging studies. At the 12-month follow-up, the patient remained asymptomatic with normal tumor markers and no evidence of recurrence on pelvic ultrasound/CT, achieving 1-year progression-free survival (PFS). Long-term monitoring continues.

## Discussion

FATWO exhibits a unique embryological origin. During female reproductive system development, the Wolffian duct guides caudal growth of the Müllerian duct, ultimately fusing with the urogenital sinus to form the lower vagina (5, 6). Under the influence of the anti-Müllerian hormone, the Wolffian duct remnants persist in structures including the broad ligament, the lateral cervical wall, and the vaginal fornix, with FATWO arising from these vestigial tissues (7). This explains the predominant localization of FATWO in the upper Wolffian system derivatives (e.g., the broad ligament), whereas mesonephric adenocarcinomas typically occur in the lower portions. The imaging characteristics of FATWO are nonspecific, which often leads to a broad differential diagnosis preoperatively. In our case, ultrasonography and CT revealed a solid, well-demarcated adnexal mass, findings that are consistent with the limited reports available. The seminal work by Matsuki et al. in 1999 first detailed the MRI features of this entity, providing valuable insights for radiologists. In their report, the tumor appeared isointense to the skeletal muscle on T1-weighted images and demonstrated heterogeneous, slightly high signal intensity on T2-weighted images, often with areas of cystic degeneration. Following contrast administration, the solid components typically showed homogeneous enhancement (8). While our patient did not undergo MRI, the solid nature and well-defined borders observed on CT align with the fundamental MRI description of a solid mass. The key radiological challenge, as highlighted by Matsuki et al., is how to differentiate FATWO from the more common entities such as a subserosal leiomyoma or an ovarian stromal tumor (e.g., thecoma or fibroma). The presence of a separate, normal ipsilateral ovary on imaging can be a crucial clue to suggest an extra-ovarian origin, such as a FATWO arising from the broad ligament. Ultimately, however, the overlapping imaging features underscore the necessity of a histopathological examination for a definitive diagnosis, which was performed in our case.

The principal differential diagnoses for FATWO include a well-differentiated endometrioid adenocarcinoma, a Sertoli–Leydig cell tumor, and a granulosa cell tumor. Endometrioid adenocarcinomas frequently originate from fallopian tubes and demonstrate a more pronounced nuclear atypia and mitotic activity (1). Although Sertoli–Leydig cell tumors share morphological similarities, FATWO lacks endocrine manifestations and may exhibit characteristic cribriform patterns (9). Granulosa cell tumors are identified by their pathognomonic nuclear grooves, although this feature is not entirely specific (10). Distinguishing Wolffian tumors from sex cord–stromal tumors reveals particular challenges, as both may express calretinin, inhibin, WT1, and CD10 while typically remaining negative for EMA and PAX8 (11, 12). Sex cord–stromal tumors typically demonstrate diffuse inhibin positivity, whereas female adnexal tumors of Wolffian origin generally show focal expression patterns. FATWO displays considerable morphological heterogeneity (solid, tubular, and cribriform patterns), necessitating combined morphological and immunohistochemical evaluation due to its nonspecific features (4, 13). The immunohistochemical profile of our case presented with strongly positive FOXL2 (+++), calretinin (++), SF-1 (++), α-inhibin (++), and vimentin (++), focal luminal CD10 positivity, and AR (+), but with negative ER, PR, EMA, GATA3, and TTF-1, aligning with the established FATWO characteristics in the literature (13, 14). This immunophenotype, in particular the strong positivity of FOXL2 contrasting with the variable expression of sex cord–stromal tumors, provides critical diagnostic support for FATWO. The dual negativity of GATA3 and TTF-1 effectively excludes metastatic carcinomas of breast, urothelial, or thyroid origin, while the hormonal receptor negativity (ER/PR) further differentiates it from hormone-responsive neoplasms. The low Ki-67 index (1%) observed correlates with the typically indolent biological behavior of FATWO.

Heatley et al. proposed, based on a UK literature review, that Wolffian tumors cannot be entirely regarded as benign lesions (15). Furthermore, there are documented cases of contralateral adnexal and pelvic–abdominal metastases occurring 2 years after fertility-sparing surgery for Wolffian tumors (16). The current literature primarily focuses on postmenopausal patients (with favorable prognosis following total hysterectomy and bilateral salpingo-oophorectomy), while the therapeutic dilemma for younger patients remains unresolved. While infracolic omentectomy, peritoneal washings, and lymph node sampling are standard for epithelial ovarian carcinomas, their role in FATWO remains undefined. Given the rarity of the tumor and its generally indolent behavior, staging procedures are typically reserved for cases with overt malignant features (e.g., tumor rupture, gross metastases, or high-grade morphology). In our case, the tumor was intact and confined to the adnexa without suspicious extra-ovarian findings, justifying a conservative approach. Nonetheless, future studies should evaluate whether systematic staging impacts the survival or recurrence risk of patients with adverse pathological features. It must be emphasized that there is a notable lack of reliable evidence with regard to the application of fertility-preserving treatments in premenopausal patients and their long-term efficacy. Although FATWO typically exhibits an indolent clinical course, its potential for aggressive progression necessitates the establishment of evidence-based therapeutic strategies.

Our targeted literature review of FATWO cases from the past decade (**Table 1**) revealed significant heterogeneity in the treatment approaches—ranging from surgery alone to multimodal therapy. Of particular note, while isolated case reports have described transient remission with adjuvant therapies (including chemotherapy, radiotherapy, hormonal agents, and targeted drugs), the definitive role of these interventions in malignant FATWO remains unestablished due to the absence of controlled clinical studies.

**Table 1:** Summary of the clinical characteristics and management in reported cases of female adnexal tumor of Wolffian origin (FATWO) from the past decade.

Author (year)	Age (years)	Child-bearing history	Clinical presentation	Primary site and tumor size (diameter: cm)	Tumor nature	Treatment category	Follow-up and outcomes
Shabnam Mashhadi (2024) (16)	35	G1P1	None	Left adnexal: 10	Malignant behavior	First: Ovarian cystectomy For recurrence: TH + BSO+ six cycles of paclitaxel (175 mg/m^2^) and carboplatin (AUC5) chemotherapy	Recurrence at 2-year follow-up	
Shuhui Hong (2018) (17)	50	G1P1	Lower abdominal pain, constipation, and increased urinary urgency	Left adnexal: 9 Right adnexal: 5	Malignant behavior	Surgery: TH + BSO	NR	
Qiuhe Chen (2021) (18)	75	G3P2	None	Right pelvis: 5	NR	First: BSO Second: TH and systemic chemotherapy: six cycles of docetaxel (80 mg/m^2^) and carboplatin (300 mg/m^2^) 3 weeks after the second surgery	Recurrence was followed up for 3 years	
Alina Seixas (2020) (19)	39	G2P2	None	Right adnexal: 11	NR	2-year conservative treatment failed (fertility preserved) Surgery: TH + BSO	No recurrence at 4 years of follow-up	
Cheng Chi (2025) (20)	64	Unclear	Persistent abdominal pain and distension	Left adnexal: 11	Malignant behavior	Surgery: TH + BSO Adjuvant chemotherapy with four cycles of paclitaxel and carboplatin (TC regimen) was administered	No recurrence at 3-month follow-up	
Ya-Qiong Du (2017) (21)	73	NR	Abdominal pain and bloating	Left ovary: 26	Benign behavior	Simple tumor resection	No recurrence at 6-month follow-up	
Tian Qiu (2017) (22)	53	NR	Abdominal distension	Left mesosalpinx: 13	NR	First: TH + BSO Second: Cytoreductive surgery + chemotherapy was performed with cisplatin intraperitoneally and docetaxel intravenously	Liver and renal failure 2 months after second surgery. Died of the disease	
Lenan Liu (2018) (23)	34	G0P0	Unclear	Left adnexal: 6	NR	Left salpingo-oophorectomy	NR	
A. Piciu (2021) (24)	43	NR	Unclear	Right adnexal: 7	Malignant behavior	First: TH + unilateral resection of the right ovarian cyst Second: Both of the annexes were resected along with the omentum and the lymph nodes in the Douglas and subhepatic region Third: Excision of the lymph nodes was performed followed by peritoneal diathermy+ six cycles of adjuvant chemotherapy given using paclitaxel and carboplatin Fourth: Cytoreduction + three cycles of carboplatin (AUC5) and etoposide 100-mg regimen Fifth: Excision of the masses + four cycles of chemotherapy with topotecan	Recurrence four times. Gave up the treatment and loss of evidence of the patient	
G. Khastgir (2024) (25)	60	G4P4	Intermittent lower abdominal pain for 2 years	Right adnexal: 9.8	NR	TH + BSO + peritoneal biopsy and lymph node sampling	No recurrence at 5 years of follow-up	
M. Goel (2025) (26)	65	NR	None	Fat (3.0 cm), rectouterine pouch (0.9 cm), and left fallopian tube surface (0.7 cm)	Benign behavior	First: Revealing a remote history (32 years prior) of a left para-ovarian cyst Second: TH + BSO	Alive without radiologic evidence of disease at 15 months. Continued surveillance with CT scans every 6 months is planned.	

*TH + BSO*, total hysterectomy + bilateral salpingo-oophorectomy; *NR*, not reported.

The management of advanced, recurrent, or metastatic FATWO poses a significant challenge due to the lack of established guidelines, with evidence confined to isolated case reports. A variety of chemotherapeutic regimens have been employed, with mixed outcomes. Combinations included paclitaxel/carboplatin (27), cisplatin/cyclophosphamide and etoposide/carboplatin (2), and cisplatin/docetaxel (22). However, the responses are often unsatisfactory and transient. For instance, a patient treated with cisplatin/oxaliplatin/docetaxel experienced adverse effects and succumbed to the disease shortly after a second surgery (22). The potential role of molecular-targeted therapy, particularly tyrosine kinase inhibitors (TKIs) such as imatinib, remains intriguing, yet inadequately defined. This interest stems from the observed KIT (CD117) immunoreactivity in a subset of FATWOs (28, 29). However, it is crucial to emphasize that KIT positivity does not consistently predict the efficacy of TKIs. As demonstrated by Wakayama et al. (28), treatment with imatinib mesylate led to only transient disease stabilization—which lasted 4 months—before progression ensued, highlighting the limitations of TKI therapy in the absence of actionable driver mutations. In contrast, more encouraging outcomes have been achieved with chemotherapy. In the same report (28), combination treatment with carboplatin and paclitaxel induced a significant response in a patient with recurrent metastatic FATWO following imatinib failure, underscoring the potential utility of platinum-based regimens in this setting.

While surgical resection remains the cornerstone of therapy, the current literature indicates that, for recurrent or metastatic disease, platinum-based combination chemotherapy (e.g., paclitaxel/carboplatin) currently presents the most supported systemic option. The use of TKIs such as imatinib should be approached with caution and may be reserved for cases with confirmed activating mutations, given the documented lack of efficacy in wild-type tumors. In the present case, considering the complete surgical excision with negative margins, the low-grade histological features, and the absence of evidence supporting a clear survival benefit from adjuvant therapy for localized disease, a decision for close clinical and radiological surveillance was made. This conservative approach is supported by the indolent nature of many FATWOs. However, given the documented risk of late recurrence, the patient will be monitored long term.

Emerging evidence suggests that FATWO may share tumorigenic mechanisms with certain ovarian cancers, particularly regarding genomic instability. Kwon et al. demonstrated that MGMT inactivation through promoter hypermethylation may contribute to the pathogenesis of FATWO (27), providing epigenetic insights into its development. Notably, Estevez-Diz et al. proposed *STK11* mutation testing for patients with FATWO, as frequent *STK11* mutations in a case series indicated potential therapeutic vulnerability to mTOR inhibitors (30). The molecular characterization of eight FATWO cases by Mirkovic et al. revealed distinct genetic alterations: *KMT2D* variants (57%), *STK11* mutations (29%, including one Peutz–Jeghers syndrome case), and *ARID1B* mutations (14%). Importantly, FATWOs lack the *KRAS*/*NRAS*, *TP53*, and *DICER1* mutations typically seen in mesonephric carcinomas or Sertoli–Leydig cell tumors, with an overall low mutational burden, suggesting divergent oncogenic pathways (31). However, it is important to note that emerging evidence suggests that tumors harboring *STK11* mutations may represent a distinct biological subset, sometimes termed “*STK11* adnexal tumors,” which could be distinguished from classic *STK11* wild-type FATWOs in future classifications. This distinction is crucial as it may have significant implications for targeted therapy and genetic counseling (32).

Our literature synthesis highlights the diagnostic dilemma for FATWO: early-stage manifestations are nonspecific, with normal tumor markers in most cases. Symptomatic presentations typically result from mass effects rather than biochemical activity. Although conventionally classified as benign, accumulating reports have described postoperative recurrences and metastases. Current evidence shows uncertain chemotherapy efficacy, leaving complete surgical resection as the mainstay treatment. Given its malignant potential, long-term surveillance remains imperative. This study has several limitations that should be considered. Firstly, the retrospective nature of the analysis may introduce inherent biases. Secondly, our immunohistochemical panel did not include *PAX8* and *WT1*, which are pivotal markers for excluding Müllerian lesions, including mesonephric and serous carcinomas. While the distinctive morphology and the existing profile (e.g., calretinin+/inhibin+/GATA3−) strongly support the diagnosis of FATWO, the addition of these markers would have provided greater diagnostic confidence and more comprehensively ruled out morphological mimics.

## Conclusions

Based on current evidence, FATWO should not be categorically considered benign. These neoplasms demonstrate concerning biological behaviors, including local invasion, recurrence, and metastatic potential. For postmenopausal patients, total hysterectomy with bilateral salpingo-oophorectomy represents the standard approach. However, the role of adjuvant therapy requires an individualized multidisciplinary team (MDT) evaluation considering pathological risk factors. Notably, the current evidence-based recommendations for fertility-age patients remain insufficient, highlighting the urgent need for high-quality studies to guide clinical practice.

## Data Availability

The original contributions presented in the study are included in the article. Further inquiries can be directed to the corresponding author.
